# Invasive Aspergillosis in Refractory Angioimmunoblastic T-Cell Lymphoma

**DOI:** 10.4274/tjh.2017.0236

**Published:** 2018-03-06

**Authors:** Prakash NP, Anoop TM, Rakul Nambiar, Jaisankar Puthusseri, Swapna B

**Affiliations:** 1 Regional Cancer Centre, Department of Medical Oncology, Thiruvananthapuram, India; 2 Regional Cancer Centre, Department of Microbiology, Thiruvananthapuram, India

**Keywords:** Lymphoma, Endophthalmitis, Aspergillus

## To the Editor,

**Figure 1:**
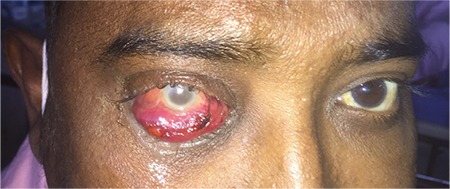
Photograph showing red conjunctival swelling over the right eye.

A 40-year-old man with angioimmunoblastic T-cell lymphoma, on palliative chemotherapy with lenalidomide at 20 mg, developed pancytopenia and progressive loss of vision and conjunctival swelling over the right eye after the second cycle (Figure 1). Brain magnetic resonance imaging with orbit demonstrated endophthalmitis. A pus sample was inoculated onto routine bacteriological media and Sabouraud’s dextrose agar (SDA) for detection of fungal pathogens. On the 4^th^ day, fungal growth was observed on SDA. The surface of the fungal colony was initially white; it turned to a blue-green color and had a powdery texture. Lactose phenol cotton blue mount showed hyaline septate hyphae with short conidiophores and vesicle-bearing chains of round conidia covering the upper half of the vesicle, suggestive of *Aspergillus fumigatus*. He was started on parenteral voriconazole, but his condition worsened and he died following severe fungal sepsis.

Orbital invasive aspergillosis is a fatal condition, often misdiagnosed, and the mortality rate remains high even after proper treatment. Patients at risk for invasive aspergillosis include patients with prolonged neutropenia, allogeneic hematopoietic stem cell recipients, solid organ transplant recipients, patients on chronic steroid therapy, and patients with HIV infection or chronic granulomatous disease [1,2]. Among patients with hematologic conditions (both benign and malignant), the duration and grade of neutropenia predict the risk of invasive aspergillosis. The incidence of invasive aspergillosis in patients with hematologic malignancies has been reported to be as high as 3.1%, with *Aspergillus fumigatus *representing the most commonly isolated species [3]. Compared to amphotericin B, voriconazole demonstrates a survival benefit, less systemic toxicity, and better tolerance by patients [4].
